# Supplementary material for: Health-related quality of life in non-alcoholic fatty liver disease: A cross-cultural study between Spain and the United Kingdom
Source: PLoS One. 2024 May 6;19(5):e0300362. doi: 10.1371/journal.pone.0300362 (PMC11073709; doi:10.1371/journal.pone.0300362)
Supplement: S1 Table — (DOCX) [file pone.0300362.s001.docx]

**S1 Table. Indirect effects of emotional function, body mass index and fatigue mediating in the association between gender and health-related quality of life**

| **Indirect effect key** | **Effect (*BootSE*)** | ***p*** | **Bootstrapped 95% CI** | |
| --- | --- | --- | --- | --- |
|  |  |  | **Lower** | **Upper** |
| G – E – Q | -0.200 (0.038) | <0.001 | -0.276 | -0.125 |
| G – B – Q | 0.002 (0.005) | 0.633 | -0.008 | 0.013 |
| G – F – Q | -0.059 (0.032) | 0.055 | -0.120 | 0.002 |
| G – E – B – Q | -0.007 (0.002) | 0.002 | -0.012 | -0.002 |
| G – E – F – Q | -0.165 (0.032) | <0.001 | -0.230 | -0.104 |
| G – B – F – Q | 0.002 (0.004) | 0.630 | -0.006 | 0.011 |
| G – E – B – F – Q | -0.006 (0.002) | <0.001 | -0.010 | -0.002 |

G, gender; E, emotional function; Q, health-related quality of life; B, body mass index; F, fatigue; *BootSE*, bootstrap standard error; CI, confidence interval.
