# Supplementary material for: Health-related quality of life in non-alcoholic fatty liver disease: A cross-cultural study between Spain and the United Kingdom
Source: PLoS One. 2024 May 6;19(5):e0300362. doi: 10.1371/journal.pone.0300362 (PMC11073709; doi:10.1371/journal.pone.0300362)
Supplement: S2 Table — (DOCX) [file pone.0300362.s002.docx]

**S2 Table. Effects of moderation by place of origin (Spain or UK) on the relationship between fatigue and health-related quality of life**

| **Place of origin** | **Effect (*SE*)** | ***t (p)*** | **Bootstrapped 95% CI** | |
| --- | --- | --- | --- | --- |
|  |  |  | **Lower** | **Upper** |
| Spain | 0.349 (0.017) | 20.536 (<0.001) | 0.315 | 0.382 |
| UK | 0.452 (0.022) | 20.650 (<0.001) | 0.409 | 0.495 |

*SE*, standard error; CI, confidence interval.
