# Supplementary material for: Health-related quality of life in non-alcoholic fatty liver disease: A cross-cultural study between Spain and the United Kingdom
Source: PLoS One. 2024 May 6;19(5):e0300362. doi: 10.1371/journal.pone.0300362 (PMC11073709; doi:10.1371/journal.pone.0300362)
Supplement: S3 Table — (DOCX) [file pone.0300362.s003.docx]

**S3 Table. Conditional indirect effect of gender (male and female) on health-related quality of life through emotional function, body mass index and fatigue**

|  | **Place of origin** | **Effect *(BootSE)*** | **Bootstrapped 95% CI** | |
| --- | --- | --- | --- | --- |
|  |  |  | **Lower** | **Upper** |
| **G – F – Q** |  |  |  |  |
| - **Effect 1** | Spain | -0.054 (0.029) | -0.112 | 0.001 |
| - **Effect 2** | UK | -0.070 (0.038) | -0.147 | 0.002 |
| - **Effect 2 – Effect 1** |  | -0.016 (0.010) | -0.038 | 0.000 |
| **G – E – F – Q** |  |  |  |  |
| - **Effect 1** | Spain | -0.151 (0.030) | -0.212 | -0.096 |
| - **Effect 2** | UK | -0.196 (0.039) | -0.276 | -0.124 |
| - **Effect 2 – Effect 1** |  | -0.045 (0.014) | -0.075 | -0.020 |
| **G – B – F – Q** |  |  |  |  |
| - **Effect 1** | Spain | -0.002 (0.004) | -0.005 | 0.010 |
| - **Effect 2** | UK | -0.002 (0.005) | -0.007 | 0.013 |
| - **Effect 2 – Effect 1** |  | -0.000 (0.001) | -0.002 | 0.003 |
| **G – E – B – F – Q** |  |  |  |  |
| - **Effect 1** | Spain | -0.005 (0.002) | -0.009 | -0.002 |
| - **Effect 2** | UK | -0.007 (0.003) | -0.012 | -0.002 |
| - **Effect 2 – Effect 1** |  | -0.001 (0.001) | -0.003 | -0.001 |

G, gender; F, fatigue; Q, health-related quality of life; E, emotional function; B, body mass index; *BootSE*, bootstrap standard error; CI, confidence interval. Bootstrapping was employed to analyse the conditional indirect effect.
