# Supplementary material for: Health-related quality of life in non-alcoholic fatty liver disease: A cross-cultural study between Spain and the United Kingdom
Source: PLoS One. 2024 May 6;19(5):e0300362. doi: 10.1371/journal.pone.0300362 (PMC11073709; doi:10.1371/journal.pone.0300362)
Supplement: Attachment — Submitted filename: Response to Reviewers.docx [file pone.0300362.s004.docx]

**Response to Editor Comments**

**Point 1:**

Dear Dr. Jesús Funuyet-Salas, this manuscript was reviewed by 2 independent reviewers and the editor. The major concerns were the lack of matching between the two patients’ groups (Spanish and UK) regarding BMI, education level and employment activity. In addition, the two patient groups were not compared regarding the severity of liver fibrosis and the presence of associated comorbidities (like diabetes mellitus, hypertension, etc..). Indeed, these concerns need major revision, please review the reviewers' comments.

**Response 1:**

Dear editor, thank you for your review. The lack of matching between the two patient cohorts regarding BMI and liver fibrosis was discussed as a study limitation. Differences in education and employment were statistically significant but not important (small effect sizes). Effect sizes were defined as: null (d < 0.2; w < 0.1), small (d > 0.2; w > 0.1), medium (d > 0.5; w > 0.3) or large (d > 0.8; w > 0.5). Only statistically significant differences with medium or large effect sizes were considered important in this manuscript. This is discussed in Page 8 lines 158-160.

In addition, the two patient cohorts were compared with respect to BMI as obesity is the metabolic condition most closely linked to NAFLD. The fact that we did not consider the effects of other metabolic comorbidities such as diabetes or hypertension has been discussed as a study limitation in Page 23 line 420. We hope the editor finds this explanation sufficient.

**Response to Reviewer 1 Comments**

**Point 1:**

Title is very relevant for holistic management of NAFLD as HRQoL is underestimated issue.

Introduction covers background knowledge, knowledge gap and specific objectives of the study clearly.

**Response 1:**

Thank you for your review and your encouraging comments on our manuscript.

**Point 2:**

In Methodology patient reported outcome CLDQ followed to search HRQoL but need to exclude other chronic functional illness associated with NAFLD eg diabetes, hypertension and these may be confounder. These confounding effect was not removed.

**Response 2:**

Thank you for this suggestion. The two patient cohorts were compared with respect to BMI. This is because obesity is the metabolic condition most closely linked to NAFLD. The fact that we did not consider the effects of other metabolic comorbidities such as diabetes or hypertension was discussed as a study limitation in Page 23 line 420.

**Point 3:**

Definition of NAFLD done only Biopsy, possibly these patients are more advanced NAFLD so quality of life more disrupted.

**Response 3:**

Thank you for your comment. Liver biopsy is currently considered as the gold standard for the diagnosis and histological assessment of NAFLD. All the participants were biopsy-proven NAFLD patients, which provides added value to the validity of the study results. In terms of liver severity, we recruited patients with no or mild fibrosis as well as those with moderate or severe fibrosis. However, this has been discussed as a study limitation in Page 23 line 419.

**Point 4:**

Result and abstract of manuscript were concordant.

**Response 4:**

Thank you for your positive comment on our manuscript.

**Response to Reviewer 2 Comments**

**Point 1:**

Funuyet-Salas et al., described Health-related quality of life in non-alcoholic fatty liver disease patients from Spain and the United Kingdom. I have the following comments: 1- Although interesting, however it is not novel. The same research question was addressed in a similar study from different European countries including Spain and UK (https://pubmed.ncbi.nlm.nih.gov/30580090/). Another recent review confirmed the same (https://pubmed.ncbi.nlm.nih.gov/32435754/).

**Response 1:**

Thank you for your review and your precise helpful suggestions that contributed to the improvement of the manuscript.

As suggested in the introduction of the manuscript, the study by Huber *et al.*, (2019) is the only one that has so far compared the quality of life of NAFLD patients in different European countries. Indeed, whilst the study included Spain, it only reported on 17 participants from Spain. Our manuscript is substantially larger and included 513 Spanish participants. We therefore believe that our study provides more accurate results on the comparison between Spain and other countries. In addition, cross-cultural comparisons were only addressed minor component of Huber *et al.,* (2019). In order to understand this issue better, we sought to go deeper into the differences in HRQoL between Spanish and UK participants as is described in Page 4 line 98.

For McSweeney *et al*., (2020), which was led by members of our team was a review of extant literature, it focused mainly on the impact of NASH-associated cirrhosis from a humanistic perspective. The literature it considered whilst useful was very different in term of design to the current study and was designed to support the development of a patient reported outcome measure suitable for use in trials of treatments for NAFLD. The study was not in any way quantitative and sought to identify issues and themes relevant to the assessment of HRQoL. Given our focus on HRQoL in NAFLD patients from a cross-cultural and quantitative approach we strongly argue that the current study and McSweeney *et al*., (2020) do not overlap at all but are complementary in terms of adding to the evidence base for NAFLD.

**Point 2:**

2- The manuscript needs language editing (e.g., “Methods: HRQoL (CLDQ) was measure in both Southern” should be written as “measured”).

**Response 2:**

Thank you for this correction. Change made.

**Point 3:**

3- In the sentence “In general, the negative impact of MODERATORS on HRQoL was reported to a greater degree in UK than in Spanish participants” what does moderator means?

**Response 3:**

"Moderator" means the mediating and moderating variables in the moderated mediation model (third study objective). Specifically, the negative effects of gender on quality of life through emotional function, BMI and fatigue were higher in UK than in Spanish participants. This has been edited to put the text in plain language (Page 2 line 42).

**Point 4:**

4- Page 10 line 99 the expression “and liver severity” is not appropriate “liver diseases severity” is more suitable.

**Response 4:**

Thank you for this correction. Change made.

**Point 5:**

5- It is preferred to add S1 and S2 Tables to the main document.

**Response 5:**

Thank you for this suggestion. Change made.

**Point 6:**

6- Please add ethics committee approval number.

**Response 6:**

Thank you. We have now added this information.

**Point 7:**

7- Page 13 line 189: the sentence “There were no important between-group differences” needs rephrasing.

**Response 7:**

Thank you for this suggestion. Change made.
