# Supplementary material for: Health-related quality of life in non-alcoholic fatty liver disease: A cross-cultural study between Spain and the United Kingdom
Source: PLoS One. 2024 May 6;19(5):e0300362. doi: 10.1371/journal.pone.0300362 (PMC11073709; doi:10.1371/journal.pone.0300362)
Supplement: Attachment — Submitted filename: Response to Reviewers.docx [file pone.0300362.s006.docx]

**Response to Reviewer 3 Comments**

**Point 1:**

I do not have further suggestions.

**Response 1:**

Thank you for your review that contributed to the improvement of the manuscript.

**Response to Reviewer 5 Comments**

**Point 1:**

Methodology: You made the NAFLD diagnosis by taking liver biopsy. Why did you do liver biopsy? Is it for your study or indicated otherwise? it should be addressed in the manuscript.

**Response 1:**

Thank you for your review and your helpful suggestions that contributed to the improvement of the manuscript. The 513 Spanish participants were part of Hepamet, the Spanish registry, which diagnoses NAFLD by liver biopsy. The 224 UK participants were part of the European NAFLD Registry, which also uses liver biopsy to diagnose NAFLD.

Liver biopsy is part of the standard of care for the diagnosis of NAFLD in both Spanish and UK patient cohorts, which allowed comparison of the data from Spanish and UK participants in this cross-cultural study. This has been addressed in the manuscript (page 24, lines 423-425).

**Point 2:**

There was a statistical significant difference between the 2 groups regarding their education level and occupation. As you know, the socioeconomic level of the patients could greatly affect their HRQoL. How could you prove that the difference you found in the HRQoL is not due to the difference in socioeconomic level?

**Response 2:**

Thank you for this comment. There was indeed a statistically significant difference between the two groups in terms of education and employment. However, the effect sizes were not important (*w* = 0.194 and -0.102, respectively), as you can see in Table 1. Effect sizes are defined as: null (*d* < 0.2; *w* < 0.1), small (*d* ≥ 0.2; *w* ≥ 0.1), medium (*d* ≥ 0.5; w ≥ 0.3) or large (d ≥ 0.8; w ≥ 0.5). Only statistically significant differences with medium or large effect sizes were considered important in this manuscript (page 8, lines 158-160).

**Point 3:**

You chose Spain and UK as distinct geographical areas. Do you think they are distinct? why didn't you choose 2 so far areas especially from 2 different continents?

**Response 3:**

The aim of this study was to compare and explore quality of life in both Southern European and Northern European cohorts of patients with NAFLD. For this reason, Spain and the United Kingdom were selected for this cross-cultural study. We believe that both countries are different not only in relation to lifestyle, socio-cultural issues or estimates of obesity, but also in terms of awareness and strategies for approaching NAFLD from a public health policy perspective, as suggested in the manuscript (see Lazarus et al., 2021).

**Point 4:**

Please consider grammatical reversion of some errors.

**Response 4:**

Thank you for this comment. The manuscript has been grammatically revised again. All changes have been highlighted in yellow.
